# Are Force Enhancement after Stretch and Muscle Fatigue Due to Effects of Elevated Inorganic Phosphate and Low Calcium on Cross Bridge Kinetics?

**DOI:** 10.3390/medicina56050249

**Published:** 2020-05-20

**Authors:** Hans Degens, David A. Jones

**Affiliations:** 1 Department of Life Sciences, Manchester Metropolitan University, Research Centre for Musculoskeletal Sciences & Sport Medicine, Manchester M1 5GD, UK; hans.degens@btinternet.com; 2 Institute of Sport Science and Innovations, Lithuanian Sports University, LT-44221 Kaunas, Lithuania

**Keywords:** single fibres, Pi, BDM, stretching, force enhancement, cross bridge cycling

## Abstract

*Background and Objectives*: Muscle fatigue is characterised by (1) loss of force, (2) decreased maximal shortening velocity and (3) a greater resistance to stretch that could be due to reduced intracellular Ca^2+^ and increased Pi, which alter cross bridge kinetics. *Materials and Methods*: To investigate this, we used (1) 2,3-butanedione monoxime (BDM), believed to increase the proportion of attached but non-force-generating cross bridges; (2) Pi that increases the proportion of attached cross bridges, but with Pi still attached; and (3) reduced activating Ca^2+^. We used permeabilised rat soleus fibres, activated with pCa 4.5 at 15 °C. *Results*: The addition of 1 mM BDM or 15 mM Pi, or the lowering of the Ca^2+^ to pCa 5.5, all reduced the isometric force by around 50%. Stiffness decreased in proportion to isometric force when the fibres were activated at pCa 5.5, but was well maintained in the presence of Pi and BDM. Force enhancement after a stretch increased with the length of stretch and Pi, suggesting a role for titin. Maximum shortening velocity was reduced by about 50% in the presence of BDM and pCa 5.5, but was slightly increased by Pi. Neither decreasing Ca^2+^ nor increasing Pi alone mimicked the effects of fatigue on muscle contractile characteristics entirely. Only BDM elicited a decrease of force and slowing with maintained stiffness, similar to the situation in fatigued muscle. *Conclusions*: This suggests that in fatigue, there is an accumulation of attached but low-force cross bridges that cannot be the result of the combined action of reduced Ca^2+^ or increased Pi alone, but is probably due to a combination of factors that change during fatigue.

## 1. Introduction

During exercise, muscle becomes fatigued, defined as a reduced ability of the muscle to generate force and power [1,2]. The lower force is largely attributable to concomitant reductions in Ca^2+^-release, that consequently will cause a lower activation of the cross bridges [3]. However, reduced cross bridge recruitment cannot be the whole story, as the loss of power is more than proportional to the loss of force [2]. Indeed, during the development of fatigue, the maximal shortening velocity also decreases and the curvature of the force–velocity relationship increases (reduced a/Po), both contributing to the loss of power during a series of repeated contractions [4,5,6].

The force during a stretch is, however, relatively maintained in fatigued muscles [6,7], further indicating that during fatigue cross bridge kinetics is altered [2]. During the development of fatigue, inorganic phosphate (Pi) accumulates in the muscle [8,9], which may inhibit the release of Pi from AM.ADP.Pi cross bridges and cause an accumulation of non- or low-force-producing cross bridges, that nevertheless are well able to resist the stretching of the muscle [10]. There is also an enhancement of force at the end of a stretch that appears independent of cross bridges and fatigue [6,11]. Thus, the loss of force and power during fatigue, but not the force enhancement during a stretch, may be attributable to a reduced number of attached cross bridges, a change in the proportion of non-force producing cross bridges, and/or altered cross bridge kinetics.

Here we investigated whether the (1) loss of force, (2) reduction in maximal shortening velocity and (3) greater resistance to stretch of fatigued skeletal muscle can be explained by changes in the distribution of cross bridge intermediates. To this end, we used (1) 2,3-butanedione monoxime (BDM), believed to increase the proportion of cross bridges which are attached but not generating force, and (2) Pi that increases the proportion of attached cross bridges generating force, but with Pi still attached, and compared these conditions with (3) the effect of reducing the activating Ca^2+^. We hypothesised that increased Pi and decreased Ca^2+^ will cause changes in single fibre contractile properties that mimic the changes seen in muscle fatigue.

## 2. Methods

### 2.1. Muscle Samples

The muscles were obtained from young male adult Wistar rats humanely killed using approved schedule 1 methods for other purposes (i.e., research that had been approved by local animal ethics committees for research or teaching). This is in accord with the generally accepted guideline of reducing animal numbers to a minimum in biomedical research. The soleus muscles were excised and small fibre bundles prepared, which were immersed in a relaxing solution (see below) containing 50% (*v*/*v*) glycerol at 4 °C for 24 h, then sucrose treated as described previously [12,13] and stored at −80 °C until use. After desucrosing they were used within one month.

### 2.2. Solutions

The relaxing and maximum activation solutions (pCa 4.5) were as described previously [12,14]. The relaxing solution contained (mM): MgATP, 4.5; free Mg^2+^, 1; imidazol, 10; EGTA, 2; KCl, 100; pH 7.0. The activating solution (pCa 4.5) contained MgATP, 5.3; fee Mg^2+^, 1; imidazole, 20; EGTA, 7; creatine phosphate, 19.6; KCl, 64; pH 7.0. A pCa 5.5 solution was made to sub-maximally activate the fibres. The 2.5-, 5- and 15-mM inorganic phosphate pCa 4.5 solutions were prepared (referred to here as Pi) by adding 2.5, 5 or 15 mM potassium dihydrogen phosphate to the pCa4.5 that had a correspondingly lower potassium chloride (KCl) concentration to ensure similar ionic strengths to the normal pCa4.5 solution. Another activating solution contained 1 mM 2,3-butanedione monoxime (BDM). These solutions cause a reduction in single fibre force by an overall reduction in activation (pCa5.5), or via inhibition of cross bridge cycling (Pi and BDM).

### 2.3. Preparation of Single Fibres

The procedures for preparing and determining the single fibre contractile properties have been described previously [12,15]. Before use, the sucrose-treated bundle was desucrosed, stored at −20 °C and used within one month. On the day of the experiment, a small bundle was separated and permeabilised for 20 min with 1% Triton X-100 in relax solution on ice. Single fibres were teased out and mounted onto a permeabilised-muscle-fibre apparatus (Aurora Scientific Inc., Aurora, ON, Canada). At the start of the experiment, sarcomere length was set with a 40x objective at 2.6 μm by Fourier transformation of the sarcomere pattern (900A, Aurora). Pilot data had shown that the optimal length (*lo*) of rat soleus fibres is at a sarcomere length of 2.60 µm. The sarcomere length was checked at regular intervals thereafter. Fibre length was measured to the closest 0.01 mm and the average of three diameters measured in solution was used to calculate the fibre cross-sectional area, assuming a circular circumference of the fibre. All experiments were carried out at 15 °C. Up to 10 contractions were performed while in activating solution with fibre length being restored to *lo* after each contraction, or after each series of 4 isotonic releases. This resulted in a more stable preparation than restoring *lo* in relax solution and subsequently reactivating the fibre [16]. In all cases data were rejected if the isometric force decreased by more than 10% over the course of the experiments or the sarcomere length had changed by more than 0.1 μm.

Force–velocity relationship: To determine the force–velocity relationship, the fibre was transferred in a random order to either pCa4.5, pCa5.5, BDM or Pi and subjected to 4 series of isotonic releases [12,14]. In each series, the force was always going down from a high to a low load, and during a series of releases the muscle fibre never shortened more than 20%. The fibre was restretched to optimal length after each series of 4 releases and allowed to develop stable isometric force again before the next series of 4 releases was given. The releases varied between 5% and 90% of the maximal isometric force before the first release (Po). Length data of the last 100 ms of each 150-ms release step were used to calculate the velocity. The force and velocity data were fitted to the Hill equation using a non-linear least squares regression (Solver, Micosoft Excel).

Rate of tension redevelopment (*K_tr_*): To assess the *K_tr_,* activated fibres were released by 20% fibre length and, after 15 ms, rapidly restretched to the original length [17].

Force increment during a 1% stretch: The extra force during a 1% length-step stretch in 1 ms during the plateau of isometric peak force was measured. The extra force during a stretch divided by the amount of stretch can be used to estimate fibre stiffness [18]. It should be noted that the stretches in [18] are much smaller than our 1% stretch. However, since the stretch amplitude was similar between fibres (1%), the force increment during stretch was used as an indication of stiffness, and the force produced per cross bridge was estimated as the isometric force produced before stretch divided by the force increment during the 1% stretch.

Steady state force following stretch: After developing steady isometric force, the fibres were subjected to a 5% stretch in 800 ms at a speed of 0.0625 *lo*·s^−1^ and were then held at the new longer length. Provided there was no evidence of damage to the fibre, both stretches and releases were repeated up to four times in different conditions. Some fibres were also stretched by 1.5% and 2.5% of *lo*, and in two fibres a passive 5% stretch in 800 ms at a speed of 0.25 and 1.0 *lo*·s^−1^ was also performed. While the steady state force following stretch (force enhancement) is ideally compared to the force in a separate isometric contraction at the final stretched length, to take into account the difference in tension as a result of the length–tension relationship [19], we compared it to the isometric force prior to the stretch. Although this most likely results in an overestimation of force enhancement, this effect is small [19] and probably less than the 2.5% reduction in force per activation we have reported previously [20]. 

Fibre type determination: To classify type I and type IIa fibres, fragments were dissolved in Leamli buffer and run on a 7% SDS-PAGE at 15 °C for 27 h. The gels were stained with the Bio-rad Silver Stain Plus kit (Bio-rad, UK) and the myosin heavy chain composition determined from the migration distance of the myosin isoforms, as described previously [15]. This was done only for fibres in which the force–velocity relationship, *K_tr_* or stiffness was established.

Data recording and analysis: Data for force and length were recorded at a sampling frequency of 1 kHz. For each *K_tr_* test, data from the point at which force started to rise following the re-stretch were fitted to a single exponential using a non-linear least squares regression (Solver, Microsoft Excel). For the rate of tension decay following a stretch the force data from the end of the stretch were fitted to a triple exponential function. To assess the distribution of cross bridges in the different conditions we used the cross bridge model by [21] with adapted rate constants, using Mathematica version 7 (Wolfram, Hanborough, UK) and compared the outcome with the actual data. The rate constants were those in [21], with some slight modifications to obtain a good fit with the data (i.e., *k*_−1_, in our model *k*_21_, was elevated from 1 to 4, *k*_−2_, in our model *k*_32_, was increased to 6, and other rate constants were adapted as indicated in the Results section.

Statistics: A Kolmogorov–Smirnov test suggested that the distribution of the data did not deviate significantly from a normal distribution. Differences between conditions were tested with ANOVAs or *t*-tests and considered significant at *p* < 0.05. If an ANOVA showed a significant effect, Bonferroni-corrected post-hoc tests were performed to locate the differences. Data are presented as mean ± SD, unless indicated otherwise.

This study was approved by the Ethics Committee of the Manchester Metropolitan University under protocol number SE161752 on 6 February 2017.

## 3. Results

### 3.1. Isometric Force

**Figure 1:**
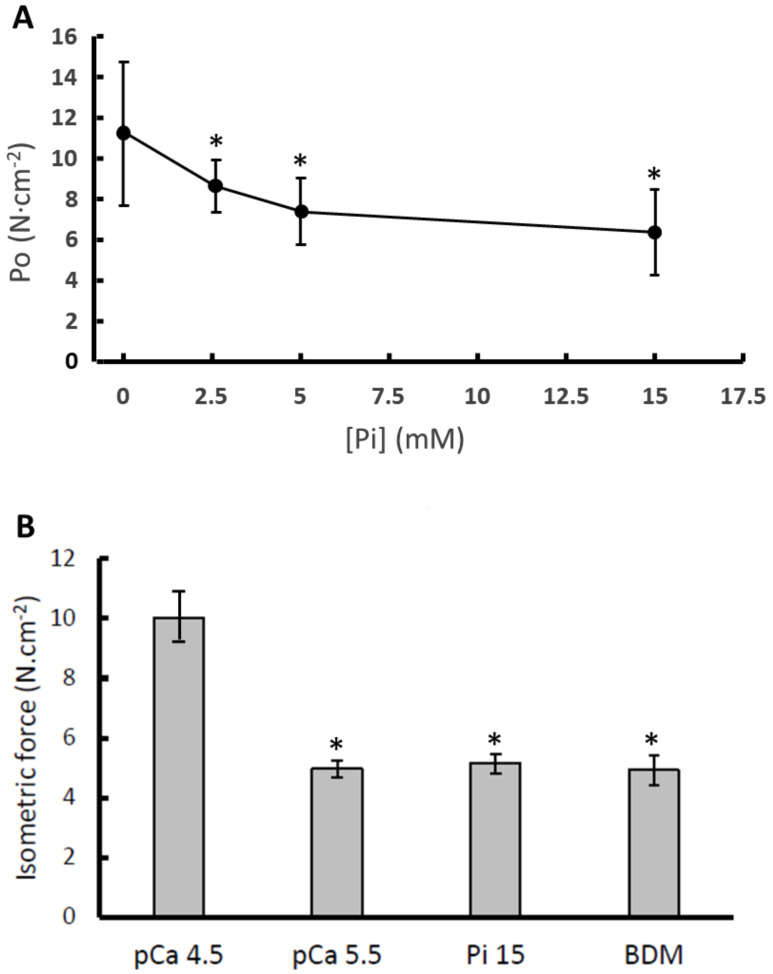
The impact of (**A**) different concentrations of inorganic phosphate (Pi) in pooled type I and IIa fibres and (**B**) low calcium (pCa 5.5), 15 mM inorganic phosphate (Pi 15) and 1 mM 2,3-Butanedione monoxime (BDM) in type I fibres only, on maximal isometric force (P_0_). Data are mean ± SD (panel A) and SEM (panel B) of 14–36 fibres. *: different from pCa 4.5 at *p* < 0.001.

In Figure 1A, it is shown that the force generated during maximal activation is decreased in the presence of Pi, with only a small difference between the force in the presence of 5 and 15 mM Pi, respectively. In Figure 1B it can be seen that the maximal isometric force was highest in the standard activating solution (pCa 4.5), was 42% lower in pCa 5.5, 43% lower in 15 mM Pi and 58% lower in 1 mM BDM (*p* < 0.001).

### 3.2. Force–Velocity Relationship

**Figure 2:**
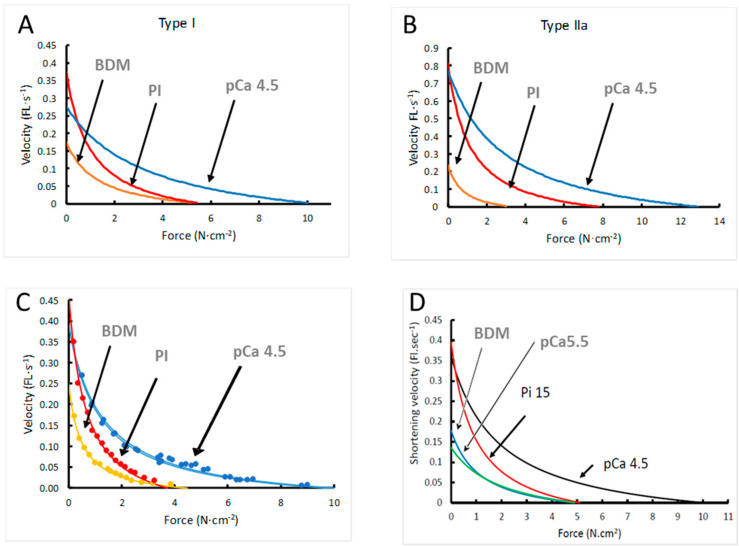
The effects of 15 mM inorganic phosphate (Pi) and 1 mM 2,3-butanedione monoxime (BDM) on the force–velocity (FL·s^−1^: Fibre lengths·s^−1^) relationship of (**A**) type I and (**B**) type IIa fibres, (**C**) an example of data of a type I fibre in which the force–velocity relationship was completed in BDM, Pi and twice in pCa 4.5 and (**D**) the results of the model. Data are shown in Table 1.

In Table 1 it is shown that 15 mM Pi and 1 mM BDM induced a reduction in the maximal isometric force in both type I and type IIa fibres (*p* < 0.01). The decrement in force induced by BDM was larger in type IIa than type I fibres (*p* < 0.001). BDM caused a reduction in maximal shortening velocity (Vmax) in both type I and type IIa fibres (*p* ≤ 0.03), and 15 mM Pi induced an increase in Vmax in type I fibres only (*p* = 0.025). The a/Po was reduced by 15 mM Pi in both fibre types (*p* = 0.001) and by BDM in type I fibres only, indicating an increase in curvature of the force–velocity relationship that would aggravate the loss of power resulting from the reduction in Po. Figure 2 illustrates the effects on the force–velocity relationship for type I (Figure 2A) and type IIa (Figure 2B) fibres, and Figure 2C shows an example of a type I fibre in which the force–velocity relationship was determined in all three conditions. Previously we showed that a low Ca^2+^ caused a decrease in Vmax and an increase in a/Po [14].

### 3.3. K_tr_

**Figure 3:**
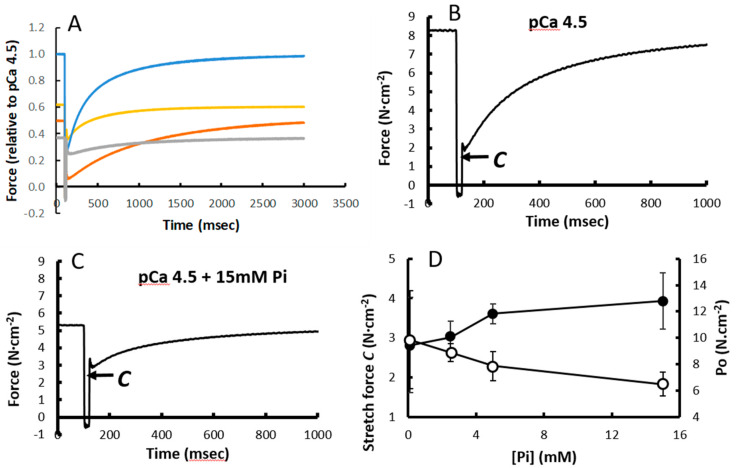
The effect of (**A**) pCa5.5, 15 mM inorganic phosphate (Pi) and 1 mM 2,3-Butanedione monoxime (BDM) on the *K_tr_*. (**B**) *K_tr_* pCa4.5 and (**C**) pCa4.5 + 15 mmPi in type I fibres, where ‘C’ indicates the force at the end of the stretch and (**D**) the force ‘C’ at the end of the stretch (solid symbols) and initial isometric force (open symbols). Data are mean ± SD of 5–12 fibres.

In Figure 3A, the *K_tr_* of type I fibres in pCa 4.5, pCa 5.5, 15 mM Pi and 1 mM BDM are shown. The time constant of recovery of force was similar in the absence and presence of 15 mM Pi (2.61 ± 0.49 s^−1^ vs. 2.65 ± 0.61 s^−1^, respectively). The force immediately after the restretch, designated as ‘*C*’ (Figure 3B), was not significantly changed in 15 mM Pi (Figure 3A,C) and 1 mM BDM, but was reduced in pCa 5.5 (Figure 3A). Figure 3D illustrates that while the isometric force decreases, *C* increases with increasing Pi.

### 3.4. Force Increment During a 1% Stretch

**Figure 4:**
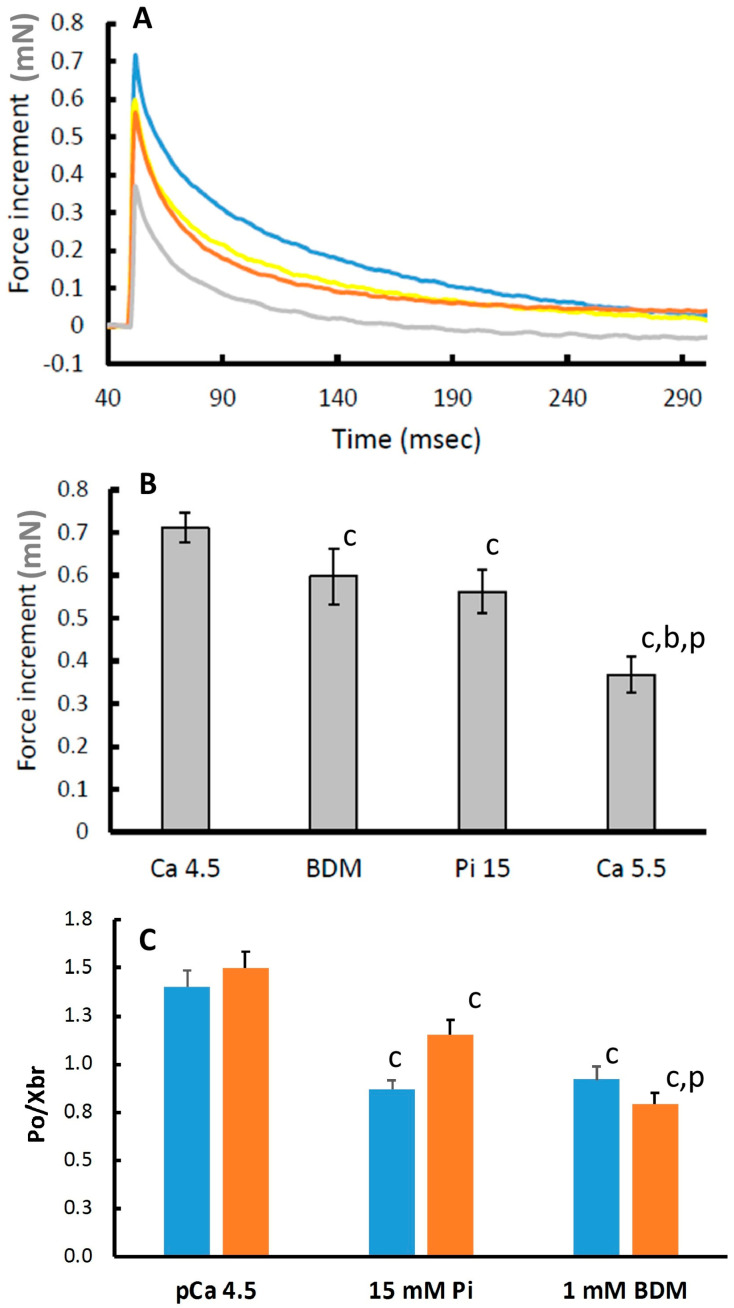
The increase in force after a 1% length-step stretch. (**A**) Blue: pCa 4.5; Grey: pCa 5.5; Brown: 15 mM inorganic phosphate (Pi); Yellow: 1 mM 2,3-Butanedione monoxime (BDM) (**B**) average force increments after a 1% step stretch. (**C**) Force per cross-bridge (P_0_/Xbr). Blue bars: Type I fibres; Orange bars: Type IIa fibres. Data are mean ± SEM of 14–36 fibres. ^c^: different from pCa 4.5 at *p* ≤ 0.002; ^b^: different from 1 mM BDM at *p* < 0.001; ^p^: different from 15 mM Pi at *p* < 0.001.

In Figure 1B, it can be seen that pCa 5.5, 15 mM Pi and 1 mM BDM all decrease the maximal isometric force by around 50%. Yet, BDM and Pi have a relatively small effect on the force increment following a 1% step stretch, while in pCa 5.5 it was reduced by around 50% (Figure 4A traces and Figure 4B average data). Assuming the rise in force during the 1% step stretch is a reflection of the number of attached cross bridges, we estimated the force produced per cross bridge by dividing the isometric force before the stretch by the force increment during the 1% stretch. The force per cross bridge was highest in pCa 4.5 and reduced in the presence of 15 mM Pi or 1 mM BDM in both type I and type IIa fibres (Figure 4C).

### 3.5. Stretching

**Figure 5:**
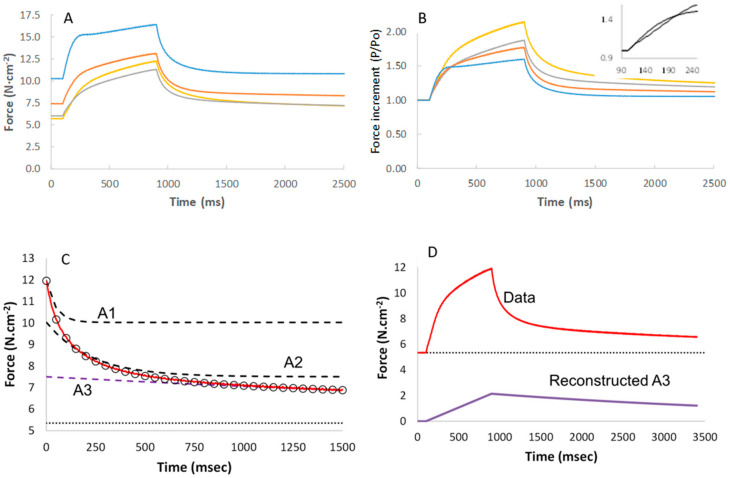
The force enhancement during a 5% stretch at 0.0625 *lo*·s^-1^ without or with 2.5 mM (orange), 5 mM (grey), or 15 mM (yellow) inorganic phosphate (Pi) in (**A**) absolute stress and (**B**) as a proportion of the isometric force before the stretch (insert: rise in force at start of stretch in expanded scale). Each curve is the average of 13–14 fibres. (**C**) Stretch relaxation fit with a three-exponential model (the open circles) to the observed relaxation (red line) with the contribution of each component (A1 fast, A2 slow and A3 very slow) illustrated by the dashed lines. (**D**) The contribution of A3 to the force increment during stretch and subsequent stretch relaxation.

**Figure 6:**
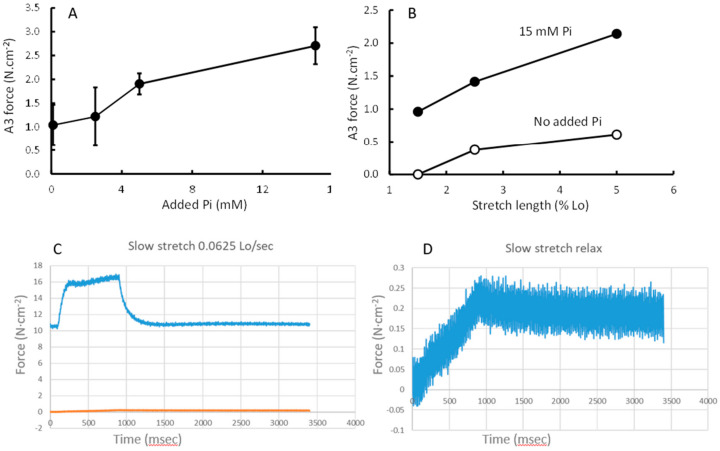
The contribution of the slow exponential component (A3) of stretch relaxation to force during a (**A**) 5% stretch at 0.0625 l *lo*·s^−1^ at different concentrations of inorganic phosphate (Pi) and (**B**) at different stretch lengths. (**C**) shows an example of a 5% stretch at 0.0625 *lo*·s^−1^ in pCa4.5 and (**D**) a similar stretch in relaxed state.

The additional force during a 5% stretch at 0.0625 *lo*·s^−1^ was similar in the presence of Pi and pCa 4.5 (Figure 5A), but the extra force as a proportion of the preceding isometric force increased with increasing Pi (Figure 5B). The relaxation after the end of the stretch could be described by a three-exponential model with a fast (A1), slow (A2) and very slow (A3) component (Figure 5C). Figure 5D shows the contribution of the A3 component to the force during the stretch and stretch relaxation. The contribution of A3 increased with increasing Pi (Figure 6A) and length of stretch (Figure 6B), but was independent of the velocity of the stretch (data not shown). Figure 6C shows a typical stretch response in pCa 4.5 that is much larger than that seen in a relaxed state (Figure 6D). It can also be seen that the force enhancement after the stretch was less (only ~0.25 N·cm^−2^ vs. ~1.0 or 2.75 N·cm^−2^) than that observed in pCa 4.5 or 15 mM Pi (Figure 6B).

### 3.6. Modelling of Cross Bridge States

**Figure 7:**
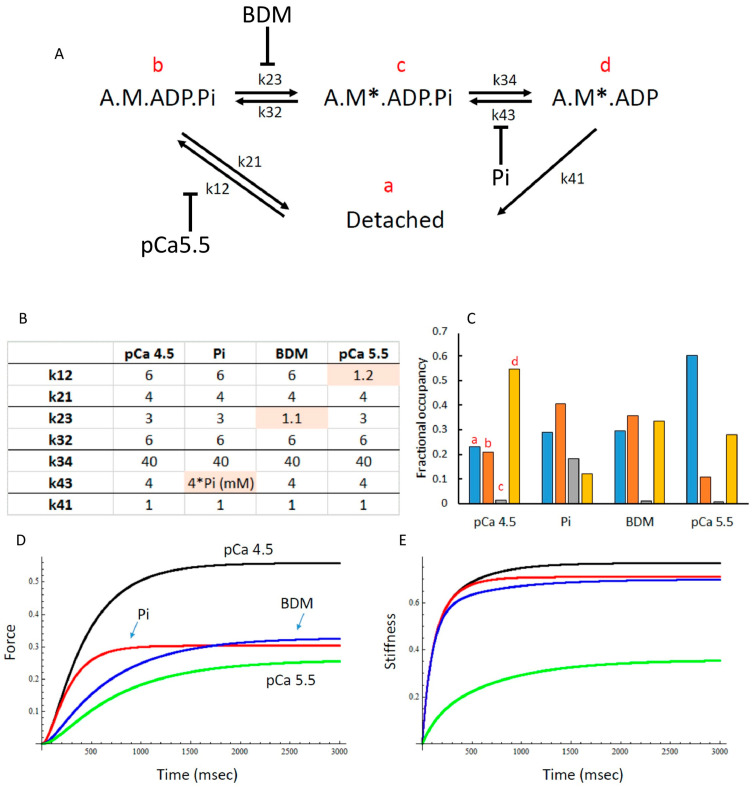
(**A**) The cross-bridge scheme used for mathematical modelling with (**B**) the rate constants in s^−1^ used and (**C**) the calculated distribution of cross bridges in pCa4.5, 15 mM inorganic phosphate (Pi), 1 mM 2,3-Butanedione monoxime (BDM) and pCa5.5. The ‘a’, ‘b’, ‘c’ and ‘d’ refer to the cross-bridge states in panel A with the same letters. (**D**) The model predicts, in correspondence with the observations (Figure 1B), a 50% reduction in force in each condition and (**E**) reduced stiffness in pCa 5.5 only (corresponding with the observations in Figure 4B).

To assess whether the changes in force and stiffness in 15 mM Pi, 1 mM BDM and pCa 5.5 could be explained by differences in the distribution of cross bridge states, we used the model shown in Figure 7A, with rate constants shown in Figure 7B. Using these rate constants, it can be seen that the proportion of detached cross bridges (indicated by ‘a’) is highest in pCa 5.5 and similar in BDM, Pi and pCa 4.5 (Figure 7C). In 15 mM Pi, but in none of the other conditions, there is significant proportion of A.M*.ADP.Pi cross bridges (indicated by ‘c’), the state just before Pi release (Figure 7C). It can also be seen that 15 mM Pi exhibits the largest proportion of non- or low force-generating cross bridges (indicated by ‘b’) (Figure 7C). The model predicted the maximal force-generating capacity (Figure 7D) and stiffness (Figure 7E) well (compare with Figure 1B and Figure 4B, respectively). In addition, the model also gave a good description of the effects of 15 mM Pi, BDM and pCa5.5 on the force–velocity relationship (Figure 2D compared with Figure 2A–C).

In Table 2 a summary of the data is given.

## 4. Discussion

Muscle fatigue can occur at many levels, ranging from central fatigue to changes in cross bridge kinetics, that may be the consequence of reduced intracellular Ca^2+^ and metabolic changes, such as a decrease in pH and accumulation of metabolites [1,2,22]. The main observations of this study are that, while lower Ca^2+^ and elevated Pi caused a reduction in force, they did not on their own mimic the changes in muscle contractile properties during the development of fatigue. More specifically, the greater resistance to stretch was not mimicked by low Ca^2+^, while the slowing was not mimicked by elevated Pi. The changes in contractile properties induced by BDM resembled fatigue most, and suggest that in fatigue there is an accumulation of attached but low-force cross bridges, that may be the result of a synergistic effect of reduced Ca^2+^, elevated Pi and other metabolic changes during fatigue, as also suggested by others [23,24].

A previous study on fast rabbit psoas fibres [25] also showed that force and stiffness were reduced in parallel with reducing Ca^2+^, while stiffness was proportionally less reduced in the presence of Pi and BDM. They also showed, like our observation in slow rat soleus fibres, that the maximal shortening velocity was increased with Pi, but reduced with BDM and low Ca^2+^. These observations thus suggest that, although type I fibres are more resistant to fatigue than type II fibres (probably related to their metabolic profile), the response to metabolites is similar in type I and type II fibres.

### 4.1. Low Ca^2+^

The proportional reductions in force, stiffness and extra force during a stretch at pCa 5.5 suggest that reducing Ca^2+^ results in an increased proportion of detached cross bridges, as indeed seen in the model calculations. Increased curvature (decreased a/Po) contributes to the decline in power during fatigue [4,5,6], and it has been suggested that this is a consequence of reduced intracellular Ca^2+^ [2]. However, pCa 5.5 was associated with an increased a/Po, as also seen before in single fibres [14] and isolated muscle preparations [26], rather than a decreased a/Po. This increase in a/Po is the result of a reduced Vmax that is proportionally larger than the concomitant reduction in *f*+*g*_1_ in the Huxley model (reflected by the *K_tr_*), also seen in previous studies at low Ca^2+^ [14,25,26]. This suggests that the effects of reduced Ca^2+^ are not only attributable to an increased proportion of detached cross bridges, but also slower cross bridge kinetics, as suggested to occur during fatigue [2]. Such a slowing of cross bridge cycling may be due to diminished cooperativity, the process whereby strong cross bridges induce a conformational change in tropomyosin, exposing more actin binding sites at low Ca^2+^ [27]. Whatever the explanation, the decrements in intracellular Ca^2+^ do not completely mimic the changes in the force–velocity relationship during muscle fatigue.

### 4.2. Effects of Elevated Pi and BDM

Here we showed, as seen previously [21,25,28,29,30], that increasing Pi causes a decrease in force that reaches an asymptote above zero, suggesting that the force-generating step occurs before Pi release. It has therefore been suggested that there is an isomerisation step between a weakly bound A.M.ADP.Pi and a strongly bound, force-generating A.M*.ADP.Pi [21,28](Figure 7A).

While it is assumed that during a *K_tr_* manoeuvre, all cross bridges are detached and the time constant of force development is a reflection of (*f*+*g*_1_) in the Huxley model [17], it is rarely considered that force immediately after the restretch (*C*) is substantially above zero. It is unlikely that any cross bridges will have remained attached after a 20% release followed by a restretch to resting length 15 ms later. This then suggests that the turnover of some cross bridges must have been extremely rapid, something that corresponds with the observation in intact muscle fibre bundles that an elevated intracellular Pi, without changes in pH and Ca^2+^ realised by a series of fatiguing contractions, was associated with faster cross bridge kinetics [11]. Here we observed that ‘*C*’ immediately after restretch was increased—at least in proportion to the maximal isometric force—with Pi and 1 mM BDM, even though maximal isometric force was 50% lower. Given that also the rate of force recovery after the restretch was not increased in the presence of Pi, this suggests that Pi and BDM induce an increase in the proportion of low-force-generating A.M.ADP.Pi cross bridges, that can rapidly detach to M.ADP.Pi and reattach. In pCa 5.5, the decrement in ‘*C*’ is proportional to the decrease in maximal isometric force, corresponding with the model calculations showing an increased proportion of detached cross bridges, and a decreased proportion of A.M.ADP.Pi cross bridges.

The inferences deduced above are further supported by measures of stiffness, indicative for the number of attached cross bridges [31,32]. It is acknowledged that the 1% step we applied was larger than that used by others to measure stiffness, and our sampling rate may have missed the real peak force after a 1-ms 1% stretch that typically occurs within 0.2 ms [18]. However, as expected, the extra force during the stretch was reduced in proportion to the decrease in isometric force in pCa 5.5, suggesting that despite the shortcomings of our approach, the procedure gives an estimate of stiffness. Here we found that the force increment in response to a 1% stretch was reduced less than in proportion to the loss of isometric force in 1 mM BDM and 15 mM Pi, suggesting an accumulation of low-force cross bridges. However, others have observed that the stiffness and force show a proportional decrement in the presence of Pi, suggesting that even in the presence of Pi, all attached cross bridges develop force [28]. The discrepancy is perhaps attributable to the fact that [28] determined stiffness during force development, while others and we studied it during stretch imposed after reaching maximal isometric force. Whatever the cause of the discrepancy, the absence of a change, or little decrease, in stiffness was also seen in intact muscle fibre bundles with elevated intracellular Pi [11], and the most likely explanation is that the maintained stiffness in BDM and Pi is attributable to an increased proportion of low-force cross bridges resistant to stretch [10,25]. This scenario can also explain the attenuated depression of force by Pi at higher temperatures, where the proportion of pre-Pi release force-generating A.M.*.ADP.Pi cross bridges are increased with increasing temperature [30], which is difficult to explain if in the presence of Pi all cross bridges already develop maximal force.

Using the cross bridge model by [21] with amended rate constants (see Figure 7A,B for details), we calculated the proportion of cross bridges in the different states in pCa 4.5, pCa 5.5, 15 mM Pi and 1 mM BDM, and were able to qualitatively reproduce the force reductions, *K_tr_* (data not shown) and stiffness responses. This indeed showed that in Pi and BDM there is an increased proportion of non-force-generating cross bridges that could resist stretch (A.M.ADP.Pi cross bridges), with, in the presence of elevate Pi, also a significant proportion of force-generating A.M*.ADP.Pi cross bridges [21,25,28,30]. An increased proportion of low-force cross bridges, due to elevated Pi, that can resist stretch, may thus explain the resistance to stretch seen in fatigued isolated mouse soleus [7] and *in vivo* human adductor pollicis [6] muscle.

Similar to previous observations in fast rabbit psoas fibres [25,29], we observed in type I fibres (type IIa showed a similar non-significant directional change) that elevated Pi induced an increase in Vmax. Here we also saw a decrease, rather than an increase in a/Po. These changes in Vmax are at first sight not explicable by an increased proportion of non-force-generating cross bridges, as they would resist shortening. However, it has been shown that the accumulation of AM*.ADP.Pi cross bridges may enhance the probability of their detachment [29]. In BDM, on the other hand, Vmax was significantly reduced in type I fibres, in line with previous observations in rabbit fast psoas fibres [25], and mimics the blebbistatin-induced reduction in Vmax, which was explained by an accumulation of AM.ADP.Pi cross bridges—similar to what we saw with BDM—that act as breaks for cross-bridge sliding [33].

### 4.3. Force Enhancement at the End of a Stretch

Here we observed that the isometric force after a stretch remained elevated. In line with previous observations *in vivo* [6] and in single fibres [10], the force enhancement was dependent on the length, but not the speed, of stretch, suggesting it was not related to cross bridges. We and others [10,19] observed that the force enhancement in relaxed fibres was negligible in comparison to the force enhancement in activated fibres, suggesting that Ca^2+^ elevates this component. Further, Pi increased force enhancement, as seen by others in intact muscle bundles [11], which contrasts with the absence of an increase in force enhancement in *in vivo* fatigued muscles [6]. Given that the reduced force and power generating capacity during fatigue is associated with an increase in Pi [8,9] and a reduction in intracellular Ca^2+^ [3], it may be that the effects of reduced intracellular Ca^2+^ and elevated Pi on force enhancement cancel each other out. A similar situation has been observed concerning the *K_tr_*, where 4 mM Pi increased the *K_tr_*, which was reduced when both H+ and Pi were elevated [24]. Here we suggest that this non-cross-bridge component is titin, which indeed can increase its stiffness in response to phosphorylation and Ca^2+^ [34]. While all these observations fit with a role of titin, another explanation could be that Pi increases the proportion of slow-detaching cross bridges (AM.ADP.Pi) that causes, similar to what was seen in BDM [19], an increased force enhancement. These explanations are not mutually exclusive.

## 5. Conclusions

Table 2 summarises the data in comparison to fatigue. Neither decreasing activating Ca^2+^ nor increasing Pi concentrations adequately mimicked the effects of fatigue on muscle contractile characteristics. Only BDM elicited a decrease of force and slower shortening velocity while stiffness was maintained, similar to the situation in fatigued muscle. This suggests that in fatigue there is an accumulation of attached but low-force cross bridges, which may be the result of counterbalancing and synergistic effects of Ca^2+^ and Pi, as seen here, and other changes during fatigue, such as a concomitant rise in H^+^ [24] and regulatory light chain phosphorylation [23]. Future work may look at the effects of a combination of a low Ca^2+^ and an elevated Pi.

## Figures and Tables

**Table 1:** The effects of 15 mM inorganic phosphate (Pi) and 1 mM 2,3-butanedione monoxime (BDM) on the force–velocity relationship in rat type I and type IIa fibres.

	pCa 4.5	15 mM Pi	1 mM BDM
**Type I**			
Po (N·cm^−2^)	10.9 ± 1.0 (25)	5.8 ± 0.64 (21) ^c^	5.8 ± 0.88 (15) ^c^
Vmax (FL·s^−1^)	0.30 ± 0.02 (25)	0.40 ± 0.04 (21) ^c^	0.19 ± 0.02 (15) ^c,p^
a/Po	0.31 ± 0.04 (25)	0.19 ± 0.02 (21) ^c^	0.24 ± 0.03 (15) ^c^
**Type IIa**			
Po (N·cm^−2^)	12.0 ± 1.0 (19)	7.4 ± 0.6 (14) ^c^	3.3 ± 0.4 (11) ^c,p^
Vmax (FL·s^−1^)	0.74 ± 0.07 (19)	0.81 ± 0.09 (14)	0.24 ± 0.03 (11) ^c,p^
a/Po	0.26 ± 0.03 (19)	0.15 ± 0.02 (14) ^c^	0.25 ± 0.02 (11)

Data are mean ± SEM (number of fibres); Po: maximal isometric force; Vmax: maximal shortening velocity; ^c^: significantly different from pCa 4.5 at *p* ≤ 0.025; ^p^: different from 15 mM Pi at *p* < 0.001.

**Table 2:** Summary of the effects of low calcium concentration (pCa 5.5), 15 mM inorganic phosphate (Pi) and 1 mM 2,3-butanedione monoxime (BDM) on the force–velocity relationship and fatigue in rat soleus fibres.

	Force	Shortening Velocity	a/Po	Muscle Stiffness
Fatigue	Decrease	Decrease	Decrease	Maintained
pCa 5.5	Decrease	Decrease	Increase	Decrease
Pi	Decrease	Increase	Decrease	Maintained
BDM	Decrease	Decrease	Maintained	Maintained
